# Studying infant mortality: A demographic analysis based on data mining models

**DOI:** 10.1515/biol-2022-0643

**Published:** 2023-07-19

**Authors:** Muhammad Islam Satti, Mir Wajid Ali, Azeem Irshad, Mohd Asif Shah

**Affiliations:** Department of Computer Science, Millennium Institute of Technology & Entrepreneurship (MiTE), Karachi, Pakistan; Faculty of Computer Science, Asghar Mall College Rawalpindi, HED, Govt. of Punjab, Pakistan; Kabridahar University, Kabridahar, Ethiopia; Division of Research and Development, Lovely Professional University, Phagwara, Punjab, 144001, India

**Keywords:** data analytics, demographic health survey, rule induction

## Abstract

Child mortality, particularly among infants below 5 years, is a significant community well-being concern worldwide. The health sector’s top priority in emerging states is to minimize children’s death and enhance infant health. Despite a substantial decrease in worldwide deaths of children below 5 years, it remains a significant community well-being concern. Children under five years of age died at 37 per 1,000 live birth globally in 2020. However, in underdeveloped countries such as Pakistan and Ethiopia, the fatality rate of children per 1,000 live birth is 65.2 and 48.7, respectively, making it challenging to reduce. Predictive analytics approaches have become well-known for predicting future trends based on previous data and extracting meaningful patterns and connections between parameters in the healthcare industry. As a result, the objective of this study was to use data mining techniques to categorize and highlight the important causes of infant death. Datasets from the Pakistan Demographic Health Survey and the Ethiopian Demographic Health Survey revealed key characteristics in terms of factors that influence child mortality. A total of 12,654 and 12,869 records from both datasets were examined using the Bayesian network, tree (J-48), rule induction (PART), random forest, and multi-level perceptron techniques. On both datasets, various techniques were evaluated with the aforementioned classifiers. The best average accuracy of 97.8% was achieved by the best model, which forecasts the frequency of child deaths. This model can therefore estimate the mortality rates of children under five years in Ethiopia and Pakistan. Therefore, an online model to forecast child death based on our research is urgently needed and will be a useful intervention in healthcare.

## 1 Introduction

One of the most widely utilized markers of a kid’s well-being and health is child mortality. Every year, millions of children below the age of 5 years die worldwide, and this demise frequency could have been avoided. Even though the global death rate for children under 5 years of age has reduced, it remains a significant public health concern. In 2020, the global death rate for children under 5 years of age was 37 deaths per 1,000 births [1]. In 2020, fewer children under 5 years of age will die than in 1990, when they perished at 93 fatalities per 1,000 lb. The Sustainable Development Goal (SDG) for children under the age of 5 years is to lower the death rate of children under 5 years of age to at least 15 per 1,000 live births by 2030 [2].

There are variances between countries in terms of mortality reduction. Children under 5 years of age have declined significantly, from 216 deaths per 1,000 live births in 1950 to 37 deaths per 1,000 live births in 2020. Despite considerable reductions in worldwide under-five mortality, many countries have high death rates. They cannot reach MDG 4’s goal to “halve the under-five mortality rate between 1990 and 2020.”

**Figure 1:**
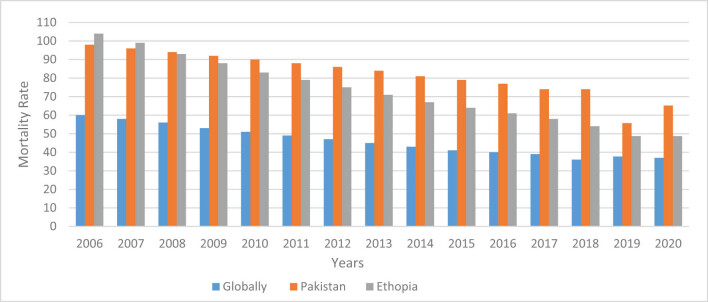
Annual mortality rate of children under the age of 5 years in Pakistan, Ethiopia, and globally.

Child mortality is a major problem in developing nations, particularly in those with middle-low incomes. Pakistan, a nation with a low health ranking, must give priority to reducing child mortality. According to the annual Human Development Index (HDI) published by the United Nations Development Program (UNDP, 2020), Pakistan is ranked 155th out of 189 countries. Pakistan has notably poor standards for children’s health. Pakistan is the third highest in the world in terms of baby, maternal, and fetal mortalities. The child mortality rate in Pakistan has reduced, from 112 deaths per 1,000 lb in 1990–1991 to 65 deaths per 1,000 lb in 2020–2021 [3]. As illustrated in Figure 1, the drop rate of mortality in Pakistan is slow as compared to that in Ethiopia.

Despite numerous healthcare sector initiatives, Pakistan’s mortality rate for children under 5 years remains lower than that of developing countries such as Ethiopia. Ethiopia had a child death rate of 104 per 1,000 births in 2006, whereas Pakistan had 98 per 1,000 births; Ethiopia now has a significant drop-off. Ethiopia’s mortality rate fell to 48.7 from 104 in 2020. However, Pakistan has yet to attain the same percentage drop in mortality as Ethiopia. Pakistan’s death frequency dropped from 98 per 1,000 live births in 2006 to 65.7 in 2020. The rapid birth of children was seen during the pandemic, where underdeveloped countries suffered more deaths than developed countries due to a lack of proper nutrition and hunger. As a result, to reach SDG4, this study examined both the country’s health data and the predictors of child mortality.

The data from many Ethiopian and Pakistani demographic health surveys are now available online, showing a decrease in childhood mortality. As a result, there is a pressing need to study and implement cutting-edge approaches and technologies to help health experts and practitioners infer knowledge and other important information from enormous datasets [3]. Big data analytics is a growing field where data mining techniques uncover interesting patterns and relationships from massive databases. Many data mining methods are freely available for use in big data analytics, and they can answer issues that standard statistical tools have previously been unable to address.

Data mining methods find application in diverse fields such as financial forecasting, intrusion and fraud detection, criminal investigation, observation of students' learning patterns, and development of educational techniques. These techniques have gained widespread usage in areas including healthcare and bioinformatics, among others, for the purpose of analysis, detection, and forecasting. Data mining techniques can detect and uncover common and unique patterns in large datasets, find a correlation between variables, and construct a prediction model. To develop models, predictive analytics approaches extract patterns from complicated, heterogeneous, vast, noisy, and incomplete data.

The main goal of this venture is to use predictive analytics approaches to present a predictive model for below-five mortality in Pakistan. The study’s primary goal is to use the findings to inform and develop intervention tactics and national initiatives to lower the number of children under 5 years of age who die in the country. Similar research articles have been investigated to achieve this goal and to identify significant factors and interactions between factors that directly or indirectly affect child mortality in local country datasets. Numerous previous studies analyzed macro-level datasets from the Ethiopian Demographic Health Survey (EDHS), the Pakistan Demographic Health Survey (PDHS), and the Pakistan Social, Living, and Standards Measurement (PSLM) and discovered significant predictors of child mortality. Numerous analytic and prediction techniques were utilized in those investigations, including logistic regression (BLR), bivariate and multivariate analysis, maximum likelihood method (MLM), Tree (J-48), and Rule-based (PART). These studies assist in selecting high-priority locations for ongoing efforts to recover motherly health and child death. This study analyses past data and forecasts whether a child will die or live in the future using advanced data mining and machine learning techniques. These predictive methods differ from more established and widely used statistical data analysis methods, relying heavily on assumptions. They have resulted in developing an improved predictive model that assists policymakers and health professionals in making timely healthcare interventions to reduce under-five child mortality.

## 2 Literature review

There is a dire need to detect the factors behind children’s death in Pakistan to reduce child mortality. There are studies examining issues affecting children’s death in Pakistan. The socioeconomic characteristics of children who died in Pakistan are determined in ref. [4]. The previous research employed the PDHS 2014–2015 dataset and analyzed 8,297 records using BLR. In addition, correlated variables were converted using principal component analysis. This study reveals that there is no difference between male and female offspring mortality rates among children under the age of 5 years and explains why. According to the study, a child’s high birth weight, extended gestation period, larger family size, the mother’s asset ownership, education, and household decision-making reduce child mortality.

Another study [5] uses BLR analysis to find and categorize indicators that demonstrate communal, monetary, and ecological factors are connected with infant death in Pakistan. “Mother’s tutoring, previous birth break, family size, child’s birth weight, birth order, Area, and breastfeeding” are all original to be significant predictors of a child death under 5 years in Pakistan. Child mortality was researched compared to other areas and found to be higher in Baluchistan. This study also noted that child mortality was much lesser when the kid was breastfed on time than when the child was not breastfed. There were no advanced predictive analytics methods or methodologies applied. Previous research has only served to indicate critical areas for intervention and identify determinants through statistical analysis.

The previous study [6] clarifies the demographic, economic, environmental, and geographic factors contributing to Pakistan’s child mortality. The dataset Pakistan social living and standards measurement, which contains 14,540 children under 8 years of age and is categorized into rural and urban zones, was used. The majority of youngsters were diagnosed with diarrheal illness. Confident children suffer from respiratory infections. The factors contributing to morbidity were identified using linear regression and maximum likelihood approaches. Child mortality can be predicted using major indicators described in the literature.

According to a study published in ref. [7], essential drivers for the death of children under 5 years of age in Pakistan were explored. Pakistan was identified as one of the five countries with the maximum death rate child under 5 years of age. This study used P-D-H-S data gathered by the National Population Studies (N-I-P-S) to determine that kids under the age of 1 year die at a high rate in Pakistan. According to the study, social factors such as awareness, media exposure, and maternal education reduce child mortality. In Pakistan, affluence, mother qualification, and media disclosure are noteworthy predictors of child death. Binary logistics and the MLM were used to estimate econometric parameters, implying that data on local zones and their inhabitants are acute for developing operative strategies to address the issues.

A method for identifying characteristics and creating a web-based application for predicting under-five child mortality in the native Ethiopian language was provided in one study [8], utilizing classification data mining techniques. J48 using EDHS data, a decision tree technique, and a PART strategy for rule induction were employed to predict child fatalities. Using the Waikato Environment for Knowledge Analysis, the best models were built (WEKA). To find the dataset’s most pertinent health predictors, we used Fisher’s score. Logistic regressions and odds ratios were used in SPSS version 20.0 to identify risk factors for child mortality. The area under the receiver operating characteristics curve, sensitivity, accuracy, and specificity were used to validate the model. The developed prediction model could be used to support at least five ongoing child health programs in Ethiopia. This article proposes that novel categorization techniques generate perfect models. The developed web-based prediction model is just for Ethiopians, and it is critical to expand this web-based application to other widely spoken languages.

The study presented in ref. [9] built a predictive analytics system to precisely estimate death rates and pinpoint the major causes of excessive child mortality. According to our research, decision trees help locate the key classification rules relating to the causes of child fatalities, and the Nave Bayes classifier has the highest average accuracy in predicting the child fatality rate (96.4%).

Predictive analytics uses historical data to determine the likelihood of a future incident. It provides an idealized perspective on what is occurring and what should be done to ensure success. It delivers the most accurate future forecast through machine learning, mathematical models, and data. Data mining is an inventive technique when faced with a massive dataset that requires prediction, analysis, or pattern identification. Machine learning is a rapidly emerging topic in computer science, information technology, and health informatics. Machine learning’s primary goal is to develop algorithms on a labeled dataset that can learn, infer, and progress over time and be utilized for prediction and analytics. Numerous data mining techniques are widely employed in various industries, and the primary healthcare industry has benefited significantly from these techniques. It provides several risk management decision support tools to enhance healthcare quality and patient safety. The healthcare sectors face significant hurdles in critical areas such as data integration, computer-aided diagnosis, and disease prediction as they strive to cut healthcare costs. Machine learning suggests several tools and techniques, algorithms, and frameworks that address these issues. This article uses various widely used prediction methodologies and data mining tools.

## 3 Methodology

Following a study of the situation and with support from earlier research, the techniques are aimed to identify the critical elements that tip to the demise of children below the age of 5 years. This was accomplished by using two case studies utilizing the demographic health dataset of Ethiopia and the demographic health survey dataset of Pakistan; these studies were mutually used to determine the demographic determinant factors. The discovery of multiple roots of child death under the age of 5 years and the necessity of urgent intervention in the healthcare arena to reduce child mortality are noteworthy. Both case studies are given in detail in the following sections, along with the determinant elements that have been investigated in each case.

### 3.1 Case study EDHS dataset

The data from the 2019 EDHS is subjected to a variety of machine learning classifiers. This research study was carried out in Pakistan from November 2020 to April 2021. It took place between November 2020 and April 2021. The identical EDHS 2019 dataset was used in this study. The CSA initially obtained the documents in Ethiopia and the United States. The information was obtained from the MEASURE DHS database [8], freely available online. The EDHS is a cross-sectional survey typical of the entire country [3], and it gives a thorough picture of demographic, maternal, and child health outcomes. The 2019 EDHS report, publicly available on the Internet, contains information on the procedures used to collect the datasets (WWW). It was possible to recover a sample of 12,654 records that satisfied the criteria. The children’s dataset was created by extracting relevant socioeconomic and demographic elements from the dataset and naming them. The infant’s survival status, which is classified as either “alive” or “dead,” serves as the study’s outcome variable. Children of “alive” mothers stayed “alive” throughout the course of the study, but babies of “dead” mothers perished once or more.

#### 3.1.1 Factors contributing to the death of children

The significant risk factors in this study include the paternal qualification, gender of a child, previous birth break, wealth index, family members, size of children at birth, birth order number, and breast milk availability. Table 1 presents the predictors used in the EDHS dataset.

**Table 1:** Predictors of children’s death in Ethiopia

Predictors	Type (C/N)	Attributes value
Place of residence	Categorical	Urban
		Rural
Breastfeeding	Categorical	No
		Yes
Preceding birth interval	Numeric	—
Parental education	Categorical	Not educated
		Primary pass
		Secondary pass
		Higher education
		Do not know
Mother occupation	Categorical	Working
		Not working
Mothers age	Numeric	—
Maternal education	Categorical	Not educated
		Primary pass
		Secondary pass
		Technical/vocational
		Higher education
		DK
Age of mother on FB	Numeric	—
Presence of diarrhea	Categorical	Yes
		No
Received family planning	Categorical	Yes
		No
Sex of child	Categorical	Male
		Female

### 3.2 PDHS dataset

The PDHS is a cross-sectional, nationally representative study [3] that assesses population, mother, and child health. The data from the 2020–2021 PDHS are used to train machine learning algorithms. It was performed in Pakistan from November 2017 to April 2018, with results published in April 2018. The PDHS 2020–2021 dataset was used in this study. A complete set of data was obtained from the MEASURE DHS database [8, which is freely accessible online]. The PDHS 2020–2021 annual report, published on the data originator’s web platform, includes data collection techniques details. The P-D-H-S used a two-stage model approach to select respondents for the study. Since sections of 12,912 homes were nominated and questioned, it was feasible to successfully interview 11,669 families in Ethiopia, excluding Gilgit Baltistan and Azad Jammu and Kashmir. Out of 1,792 households chosen, 1,597 families and 974 dwellings were cross-examined and questioned, according to the results of the Jammu and Kashmir survey.

The dataset was analyzed for pertinent socio-economic and demographic factors. The outcome variable in this study is the child’s persistent status, which is characterized as either living or dead. Alive mothers were those whose children were “alive” throughout the study, whereas the “dead” mothers were those whose infants died one or more times throughout the study.

#### 3.2.1 Risk factors associated with children’s death

The key risk factors in this study include the mother’s qualification, area, previous birth break, wealth index, family counts, size of children at delivery, and breast milk availability. Table 2 represents the predictors used in the PDHS dataset.

**Table 2:** Predictors of child mortality in Pakistan

Predictors	Type	Values
Region	Categorical	Punjab
		Sindh
		KPK
		Baluchistan
		GB
		ICT
		AJK
		FATA
Mother education	Categorical	Not educated
		Primary
		Middle
		Secondary
		Higher
Wealth index	Categorical	Poor
		Middle
		Richer
		Richest
Family members	Numeric	—
Preceding birth interval	Numeric	—
Birth order number	Numeric	—
Breastfeeding		Yes
		No
Size of children at birth	Categorical	Massive size
		Bigger than average
		Average
		I am not sure
		Smaller than the normal
		Very little

### 3.3 Experimental plan and model building

In SPSS version 20, 12,000 data records with 9 attributes are imported into the dataset. Data are cleaned using preprocessing techniques by deleting duplicate records from the dataset. The lost values are ascribed using predictive mean matching (P-M-M), a popular technique for dealing with missing data. The property also includes categorical values encoded into numerical values, such as region, which has eight category values such as Punjab, Sindh, and others. As a result, we allocated unique values to each province, such as 0 for Punjab and 7 for Fata. The same procedure was used to change the other properties.

**Figure 2:**
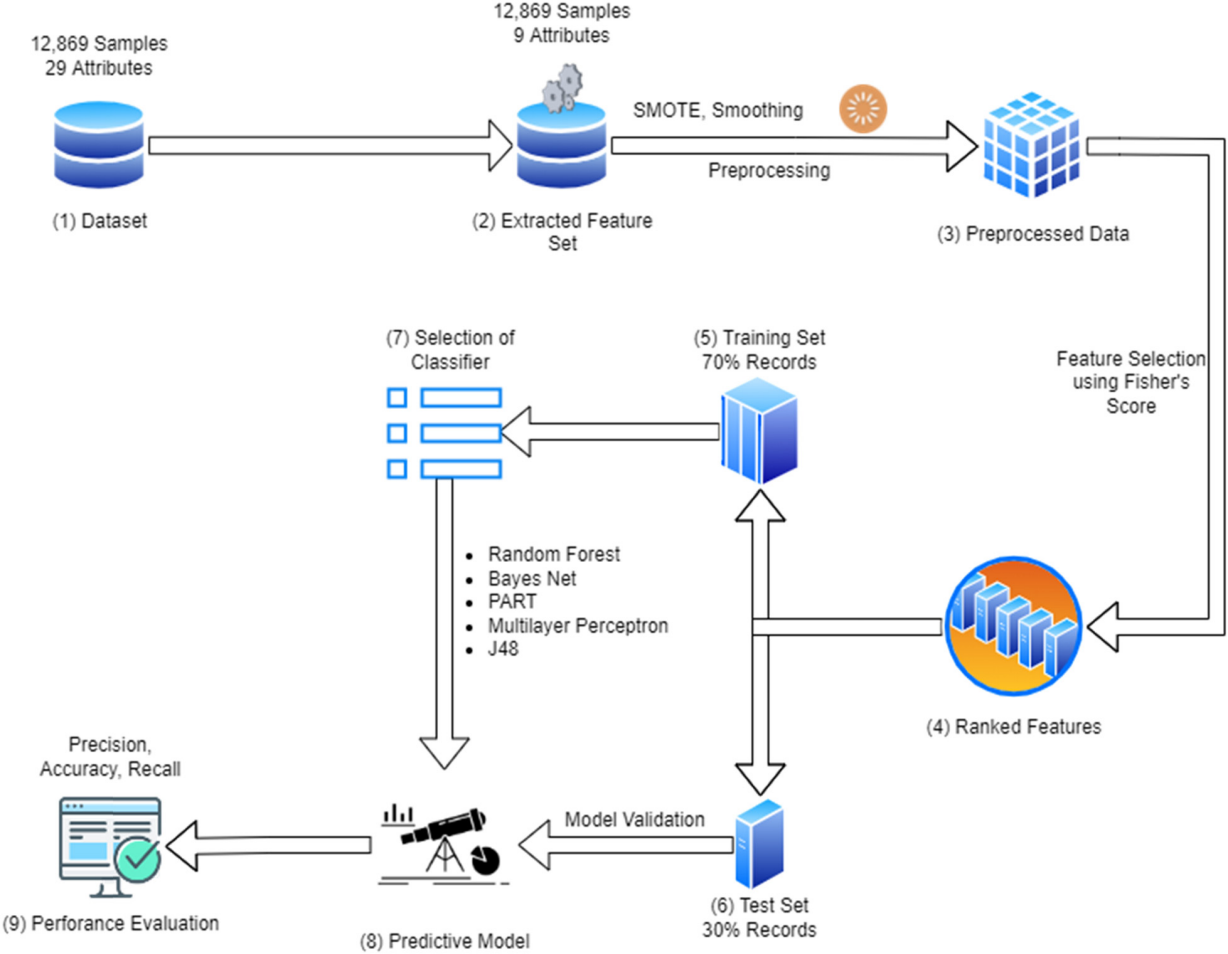
Mortality prediction model of the under-five child (MPM-UFC).

The WEKA 3.8 tool was fed the improved dataset. The refined dataset is divided into “alive” and “dead.” Moms with alive children are found in the alive clusters, while mothers with one or more deceased children are seen in the dead collections. Meanwhile, both subgroups’ data sizes are unbalanced. If the data are used as-is, the classifiers will not function well because there will be no specific proof of whether they are dead or alive or biased. The synthetic minority and oversampling technique analysis is used to equilibrium the class labels. This technique adds the records and labels synthetically by using three or more records to add one record. The sampling error is reduced to a minimum using the method described earlier. Pruning procedures apply to data that are inconsequential by cleansing the rules. Cross-validation is utilized to oversee the association and determinants’ 95% split. Figure 2 depicts the mortality prediction model of the under-five child (MPM-UFC) model.

The data are arranged using supervised classification, which uses predefined classes. The categorization uses the designated class to organize features in gathered information previously associated with class labels. Classifiers learn by training on a group of objects and then building a model to categorize the new objects [9].

A decision tree is commonly used to classify labels in the healthcare area. In the J48 decision tree classifier, a tree-like graph is used. A decision tree is a regularly utilized classifier for ineffective research analytics and determines conditional probabilities [10,11]. Pruning rule-based classification tree is the second classifier used to classify the labels (PART). The binary tree created by the decision tree J48 is a practical classification problem. When a tree is constructed, the labels are classified [12]. The J48 can be ranked in two ways: using rules developed from them or a decision tree.

The random forest was also used to categorize the classes’ labels to see which classifier performed the best on the dataset. The random forest extracts the subsections from the dataset and bootstraps a new dataset of a similar size. The bootstrap dataset contains trees built by decision trees, each taking its input. A poll of all trees assigns the new input to the class label. The maximum nominated class label is obtained through J48 for the fresh input. This classifier’s procedure selects a sample randomly. As a result, some samples may be overlooked [13].

(1) \hat{f}=\frac{1}{{\rm{Bag}}}\mathop{\sum }\limits_{b=1}^{{\rm{Bag}}}{f}_{b}(\mathop{x}\limits^{`}).]

Utilizing the aforementioned formula, bagging was calculated. The bag illustrates how bagging is repeated. FB is a classification tree that has been trained on the training set.

In the healthcare industry, the Bayesian network (Bayes Net) classifier is also used to identify important determinants and confirm the posterior probability by using the prior probability and new data. It also performs exceptionally well in data analytics and generates the most precise classifier outputs [14,15].

(2) {P}_{{\rm{B}}}({x}_{1},{x}_{2}\cdot \cdots {x}_{\Pi })=\mathop{\prod }\limits_{\dot{l}=1}^{n}{P}_{{\rm{B}}}=({x}_{i}|{\pi }_{i})=\mathop{\prod }\limits_{\dot{l}=1}^{n}\theta \frac{{x}_{1}}{{n}_{i}}.]

The sample dataset also used a multilayer perceptron for classification. It is a multilayer feed-forward artificial neural network based on nodes. The layers of the network are completely interconnected. Input nodes represent a variety of attributes. The activation function is then applied to a linear combination of input with weights W and bias B [16]. The following is the formula for the activation function in multilevel perceptron.

(3) f({z}_{k})=\frac{1}{11+e-{z}_{k}}.]

By using the “Fisher’s score” data mining technique, one of the most effective and fruitful data mining techniques, the dataset’s most significant socioeconomic and demographic health factors are extracted. High-dimensional data necessitated a huge storage capacity and a high processing cost. As a result, lowering the dimension enhances learning performance [17]. This algorithm is mostly used to reduce nonrelevant features. However, removing irrelevant characteristics is insufficient to achieve optimal perceptions and patterns. The *K*th. Fisher’s score can be used to calculate *S*_*i*_, which is

**Figure 3:**
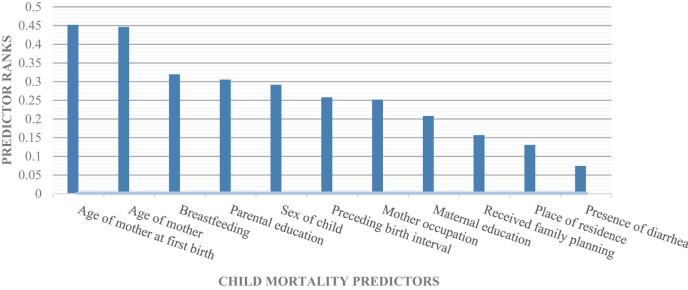
Child mortality predictors ranked set using Fisher’s score algorithm.

(4) {S}_{i}=\sum {n}_{j}({\mu }_{{ij}}\left-{\mu }_{i})2\sum {n}_{j}\times {p2}_{{ij}},]

where *i* is the mean of the *i*th feature, *ij* and *ij* are the mean and variance of the *i*th feature in the *j*th class, respectively, and *nj* is the number of occurrences in the *j*th class. In addition, the score eliminates any unnecessary elements, which are widely employed in data analytics. Figure 3 presents the traits that Fisher’s score was used to retrieve. Weka 3.8 was used to perform the preprocessing.

The property with the strongest link with the target variable is chosen in data mining. It assigns a ranking to features that influence the IG findings. Figure 3 depicts the ranking of traits according to their values.

The following evaluation measures are used to assess the classification outcomes of various algorithms. Precision, recall, and accuracy are some of the terms used to describe various metrics.

The predictive values are positive and are called predictive positive values (PPVs). The ratio of all true PPVs to the total positive predictive values is known as precision (PPV). Precision is an integer between 0 and 1. The maximum predictive value, or best value, is shown by a precision of 1, whilst the lowest predictive value, or worst value, is indicated by precision of 0.

(5) {\rm{Precision}}=\frac{{\rm{TP}}}{{\rm{TP}}+{\rm{FP}}}.]

The precision calculation is represented by equation (5) based on the true-positive and false-positive values.

The classifier’s performance is evaluated using its accuracy. The percentage of truly recognized observations to all observations is known as the truly recognized observations to all observation’s percentage. Its value is between 0 and 1. The maximum accuracy value, or best value, is indicated by a precision of 1, while the lowest accuracy value, or worst value, is shown by a precision of 0.

(6) {\rm{Accuracy}}=\frac{{\rm{TP}}+{\rm{TN}}}{{\rm{TP}}+{\rm{FP}}+{\rm{FN}}+{\rm{TN}}}.]

The accuracy calculation formula is represented by equation (6). False-positive values are designated by FP, false-negative values by FN, true positive values by TP, and true negative values by TN. False-negative values are also marked by FP.

Another word that comes to mind is “sensitivity.” It is the proportion of positively sampled positive values to all positively classified positive values. A recall rating between 0 and 1 denotes a poor recollection, whereas a recall value of 0 denotes a strong recall.

(7) {\rm{Recall}}=\frac{{\rm{TP}}}{{\rm{TP}}+{\rm{FN}}}.]

### 3.4 Experiments and results

The training and testing portions of the labeled dataset are split 70:30. Weka employs predictive modeling. Seventy percent of the survey dataset has been used to train the model, while 30% of survey datasets are used to test it initially. Before executing the modeling on the test set, cross-validation is also performed. The results of various classification models are displayed below. Table 3 demonstrates the classification of algorithm accuracy and analysis with nine predictors using the EDHS dataset.

**Table 3:** Classification and algorithm analysis with nine predictors using EDHS dataset

Algorithms	Precision	Accuracy (%)	FP rate	TP rate	Recall
Random forest	0.978	97.83	0.075	0.976	0.976
Bayes net	0.951	95.68	0.085	0.947	0.947
PART	0.931	93.99	0.097	0.930	0.930
Multilayer perceptron	0.847	85.15	0.221	0.842	0.842
J48	0.933	94.16	0.095	0.932	0.932

The Bayes Net, the best-performing classifier, achieved 97.83% accuracy by combining nine features. J48 has shown better performance with the same features and records in the literature. The Fisher’s score algorithm is run to a similar set of parameters, and the procedure returns the high-ranked features listed in Table 4.

**Table 4:** Feature sets are evaluated based on how much information they provide

Predictors	Fisher’s score value
Breastfeeding	0.3196
Age of mother	0.4463
Mothers age at FB	0.4521
Sex of child	0.3916
Received family planning	0.2568
Presence of diarrhea	0.1743
Parental education	0.3055
Mother occupation	0.2517
Preceding birth interval	0.2579
Place of residence	0.1306
Maternal education	0.2078

The attribute “mother age at first birth” had the highest score, around 1, indicating the highest importance for the correctness and strongly connected with the label, whereas “presence of diarrhea” received the lowest value. Thus, we applied the same classifier Bayes Net, Random Forest, j48, PART, and multilayer perceptron to eight top-ranked features. The Bayes Net outperformed with an accuracy of 95.68%, which is nearly equal when we utilized nine attributes. We can obtain the same accuracy with a smaller number of characteristics. The classification results for the eight qualities are listed in Table 5.

**Table 5:** Classification and algorithm analysis with nine predictors using the EDHS dataset

Algorithms	Precision	Accuracy (%)	FP rate	TP rate	Recall
Random forest	0.945	94.44	0.077	0.944	0.944
Bayes net	0.951	95.68	0.085	0.947	0.947
J48	0.932	92.94	0.099	0.932	0.929
Multilayer perceptron	0.850	83.64	0.241	0.836	0.836
PART	0.934	93.18	0.100	0.929	0.932

On the EDHS dataset, the aforementioned results were obtained. These evaluation measures have never been utilized before in the literature. As a result, this study analyses precision, recall, true predicted rate, and erroneously predictive rate. Then we took the PDHS dataset and applied the classifiers mentioned earlier before applying the same Fisher’s score technique and the same eight features. The best classifier results in the PDHS data were reached using the same “Bayes Net” technique, which had an accuracy of 97.40%. The outcomes of the classifier and their assessment results metrics are shown in Table 6.

**Table 6:** Accuracy and analysis of classification algorithms using PDHS dataset

Algorithms	Precision	Recall	TP rate	FP rate	Accuracy (%)
Random forest	0.942	0.942	0.942	0.058	94.16
Bayes net	0.976	0.974	0.974	0.037	97.40
J48	0.960	0.959	0.959	0.042	95.87
Multilayer perceptron	0.779	0.779	0.779	0.221	77.94
PART	0.960	0.959	0.959	0.042	95.89

### 3.5 Discussion

In comparison to wealthy nations, developing nations have a higher death rate per 1,000 births, making it impossible for them to meet SDGs. This study tries to identify the elements that influence the mortality of young children in Ethiopia and Pakistan. In this study, predictive analytics techniques will be employed.

Prediction in data is becoming increasingly prevalent in health care because there is a persistent need to forecast undiscovered patterns, linkages between variables, outcome labels, and other essential information in health care data [18]. Data mining provides efficient methods and other advantages, such as the diagnosis and identification of diseases. It helps the research community by leveraging and assisting in developing successful interventions in policies and maintaining individual health profiles in the development of drug recommendation systems [19,20].

The age of the mother at her first delivery, the mother’s age, breastfeeding, parental education, the child’s gender, the time between births, the mother’s occupation, her maternal education, whether she used family planning, where she lived, and whether she had diarrhea were all found to be predictors of infant death. In the majority of trials, there was a direct link between breastfeeding and lower infant mortality. Table 4 further emphasizes the significance of breastfeeding by demonstrating that it is the factor that Ethiopian and Pakistani demographic studies have found to be most effective in lowering infant death. Early breastfeeding reduces a child’s risk of dying, according to the research community [21,22]. Other research studies [23,24,25] have discovered a link between low birth weight, inadequate family planning, and the absence of a birth internal gap, and an enlarged risk of kid death. According to our research in Pakistan, children whose mothers have never utilized family planning services are less likely to pass away than children whose mothers have used [26,27,28]. Figure 3 shows that maternal age at first birth, maternal education, and the mother’s work are all important predictors of child mortality, especially in low- and middle-income nations like Pakistan. Figure 2 demonstrates the high correlation between demographic and socioeconomic factors and the target labels of dead and living. These factors detect from machine learning and ontologies developed that can help identifies the prominent indicators these findings agree with those of an Ethiopian investigation [29,30,31,32], which found similar results.

The study identified and ranked the factors highly connected with childhood mortality. We identified the demographic predictors of child mortality by applying statistical methods to survey data from the EDHS and PDHS, as shown in Table 6. In addition, this research will aid academics and practitioners in identifying and forecasting children at an upper risk of death. This research included the opinions of domain experts and compared them to the previous work on liver cancer prediction, tuberculosis prediction, and other performance predictions in the literature. In addition, this study will aid health professionals in improving facilities for teaching mothers and taking preventative measures to decrease demographic mortality, both of which benefit the public.

## 4 Conclusions and future work

Based on the survey datasets from Ethiopia and Pakistan, two case studies were completed. To identify the traits that are connected to child mortality. The study’s findings show that a number of variables, including the mother’s age at her first birth, her age at subsequent births, breast-feeding, parental qualification, the kid’s gender, the time between births, the mother’s occupation, her maternal education, her use of family planning, her residence, and the presence of diarrhea, are directly linked to children’s mortality.

They employed the Bayesian network, J48, PART, random forest, and multilayer perceptron in prior studies using their EDHS dataset. According to their findings, they produced a superior result with the Bayesian network than with other classifiers. Previously, only the J48 and PART classifiers were utilized in the research. J48 outperformed the other two approaches in this comparison. In this study, Fisher’s score was employed in conjunction with an advanced feature selection technique to provide eight top-ranked features out of a total of nine features that had previously been used. In Table 6, we demonstrate that we could attain higher accuracy by using the same classifiers on the PDHS and EDHS datasets with eight characteristics.

The algorithms employed in this study are extremely understandable and allow for easy clarification of the prediction outcome. As a result, the outcome was determined to be extremely favorable in terms of intervening in child health policies. Following this, future studies will involve developing an ideal and more general model that will encompass a broader range of demographic, socioeconomic, and genomic aspects and then combining the findings of data mining, a prediction approach, with the results of a knowledge-based system. There is a critical need to develop a powerful tool to predict child death by utilizing our data, which will serve as a beneficial intervention in the healthcare arena to support physicians in their decision-making.
